# 便携式微型液相色谱仪的研制

**DOI:** 10.3724/SP.J.1123.2021.06029

**Published:** 2021-09-08

**Authors:** Qiang FU, Limin YANG, Qiuquan WANG

**Affiliations:** 1.厦门大学化学化工学院, 谱学分析与仪器教育部重点实验室, 福建 厦门 361005; 1. College of Chemistry and Chemical Engineering, Xiamen University, the MOE Key Laboratory of Spectrochemical Analysis and Instrumentation, Xiamen 361005, China; 2.广州汇标检测技术中心, 广东 广州 510700; 2. Guangzhou Huibiao Testing Technology Center, Guangzhou 510700, China

**Keywords:** 便携式微型液相色谱仪, 大推力注射泵, 毛细管整体柱, 紫外-可见/荧光两用微型检测器, portable micro-liquid chromatograph (*p*-μLC), large-thrust syringe pump, capillary monolithic column, dual-functional ultraviolet-visible/fluorescence micro-detector

## Abstract

该工作报道了一种自行设计研制的便携式微型液相色谱仪(portable micro liquid chromatograph, *p*-μLC)。*p*-μLC集成了二元大推力注射泵作为流动相驱动装置、毛细管整体柱为分离介质和紫外-可见/荧光两用流通池为在线检测单元。自行设计研制的二元大推力注射泵可以实现等度/梯度洗脱和流动相再装填功能,可控流速范围在0.025 μL/min到5.6 mL/min之间;自制的甲基丙烯酸酯C-18有机聚合物毛细管整体微柱可实现自有机小分子至生物大分子的分离;自行研制的光纤式紫外-可见/荧光两用流通池,可以通过光纤导入来自光源的紫外光和可见光,并采集透射光和与入射光反方向射出的荧光信号,流通池内使用自聚焦透镜和全反射光导毛细管等器件提高通光效率和吸收光程;两用流通池通过光纤分别连接由大功率发光二极管/脉冲氙灯光源和微型光栅光谱仪所组成的检测装置进行在线吸收和荧光光谱检测,检测波长范围为220~700 nm。*p*-μLC采用整体手提箱式结构,流路模块、检测模块等位于下主箱体中,采集、控制模块等位于上盖中,全重不超过8 kg。仪器由装载了自编控制采集软件的内置平板电脑进行控制和数据采集。使用自行制备的甲基丙烯酸酯C-18有机聚合物毛细管整体柱,在等度洗脱模式下,在*p*-μLC上分离了烷基苯化合物混合样品,其分离检测效果可以与商品化大型HPLC仪器相媲美。

## Abstract (en)

Portable analytical instruments find extensive application in on-site examination because of their significant advantages: these instruments are convenient and easy-to-carry, leading to high time-effectiveness, and involve low reagent consumption. We report a portable micro-liquid chromatograph (*p*-μLC) that was designed and fabricated in our laboratory. The *p*-μLC integrates homemade dual large-thrust syringe pumps for delivering the mobile phase, a capillary polymer monolithic column as the stationary phase for the separation of the target analytes, and a specially designed dual-functional optical-fiber microflow-cell for online detection. The dual-thrust syringe pumps can realize isocratic and/or gradient elution as well as reloading of the mobile phase, with flow rates ranging from 0.025 μL/min to 5.6 mL/min and the maximum working pressure of 4.5 MPa. The polymethacrylate based C-18 monolithic column facilitates the separation of small organic molecules and biomacromolecules. A homemade high-power light emission diode (LED) light source and a modified xenon flash lamp are assembled as the light source module. The dual-functional detector consists of an optical fiber microflow-cell with a self-focusing lens and a light-guiding capillary, light source module, and a small-sized grating spectrometer with an output wavelength range of 400-680 nm for the LED light source and 220-700 nm for the xenon flash lamp, enabling online detection of the absorption and fluorescence spectra of the analytes from 220 to 700 nm. A bifurcated optical fiber bundle is prepared and used to connect the light source, microflow-cell, and grating spectrometer so that the incident light leading-in and the fluorescence/scatting light leading-out can be realized simultaneously. The junction end of the bifurcated optical fiber bundles connects to one end of the light path of the microflow-cell, and a straight-through optical fiber connects another end of the microflow-cell. In the UV-Vis absorption mode, the straight-through optical fiber reads out the transmitted light, while in the fluorescence mode, the excitation light beam from the light source irradiates the sample solution in the flow-cell via one branch of the bifurcated optic fiber bundles. The fluorescence leading-out via the other branch of the bifurcated optical fiber bundles in the opposite direction of the excitation light beam is read out by the spectrometer. All the large-thrust syringe pumps and flow-path, capillary monolithic column, and optical fiber mediated flow-cell detection as well as controlling modules are installed in a suitcase with a total weight of less than 8 kg. The *p*-μLC is powered by DC 12V 3A or 18650 lithium battery pack and controlled by a panel computer with a custom-built windows-based chromatography workstation software for data acquisition. When using the home-made polymethacrylate based C-18 monolithic capillary column (530 μm ID×200 mm in length), the mixed alkylbenzenes can be separated and detected in an isocratic elution mode. The separation efficiency is comparable to that obtained with a commercially available HPLC.

液相色谱(liquid chromatography, LC)是涉及复杂样品分离分析的核心技术之一^[1]^。近年来,伴随着现场和绿色低能耗分析检测以及灵活多变的联用技术的色谱偶联需求,现有大型LC仪器微型化便携式的趋势愈发凸显。但是,为了提高色谱分离效能而配备细颗粒固定相色谱填充柱的LC要求使用高压柱塞式机械泵来克服随之而来的流动相传输阻力,在一定程度上制约了LC仪器小型化便携化研究的快速发展。尽管如此,随着电子、光学和微加工技术的进步和毛细管整体色谱固定相的出现以及相关微型化检测技术的不断发展,便携式小型化/微型化LC仪器不断涌现。目前已报道的小型化/微型化便携式LC主要有以下几类:1)早期的小型化LC仪器主要是沿袭传统LC仪器结构框架,使用常规色谱柱,对柱塞机械泵和检测器等部件进行小型化。这类仪器的体积和重量虽然比实验室传统LC仪器小,实现了可移动,但距便携式微型LC的要求仍有距离^[2,3,4,5,6]^; 2)采用压缩气体^[7,8]^或热膨胀泵^[9]^驱动流动相为LC微型化提供了可能性;3)使用电渗泵、毛细管微柱和微型检测器,可以使仪器达到较高的微型化程度^[10,11,12,13]^; 4)使用高压注射泵、毛细管整体柱和微型检测元件,制作便携式微型LC仪器,因其稳定可靠,已成为了便携式微型LC仪器发展的主流^[11,12,13,14,15,16,17]^。

自2003年开始,我们课题组着手研制便携式微型液相色谱仪(portable micro LC, *p*-μLC)并制作了首台*p*-μLC^[14]^。该仪器以注射泵驱动流动相、自制毛细管整体柱作为固定相,使用自行研制的紫外-可见吸收/荧光两用流通池-光纤光谱仪进行检测,仪器总重轻至11 kg。仪器采用了模块化结构,主要部件包括注射泵、自主设计研制的光纤式紫外-可见吸收/荧光两用微型流通池^[18]^及配套使用的光纤组件^[19]^、毛细管聚合物整体柱^[20,21]^和可调式色谱柱-流通池安装架^[22]^、微型光栅光谱仪和发光二极管光源、卤钨灯光源、手动进样阀、平板控制电脑等。仪器原理流程如图1所示:流动相由注射泵驱动,从进样阀注入的样品进入毛细管整体柱进行分离,样品中的不同物质依次被洗脱进入流通池;光源发出的光由光纤导入流通池,根据不同光纤连接方式实现紫外-可见吸收或荧光检测两种模式,透射光/荧光经由光纤导入微型光栅光谱仪进行检测,检测信号经计算机处理得到相应的色谱图,整个过程可以通过触摸式平板电脑进行控制。

针对前期研制的*p*-μLC注射泵推力不足流量不够稳定、微型检测元件设计不够合理、流通池内部组件安装定位困难等问题,我们对*p*-μLC进行了不断的改进^[23]^。在升级*p*-μLC过程中,我们重新设计研制了新的紧凑型大推力注射泵和新的检测器流通池结构以及光纤组件,配备了大功率LED/脉冲氙灯组合光源等关键部件,优化了仪器结构,进一步提升了*p*-μLC的性能、减小了仪器体积和重量。

**图1:**
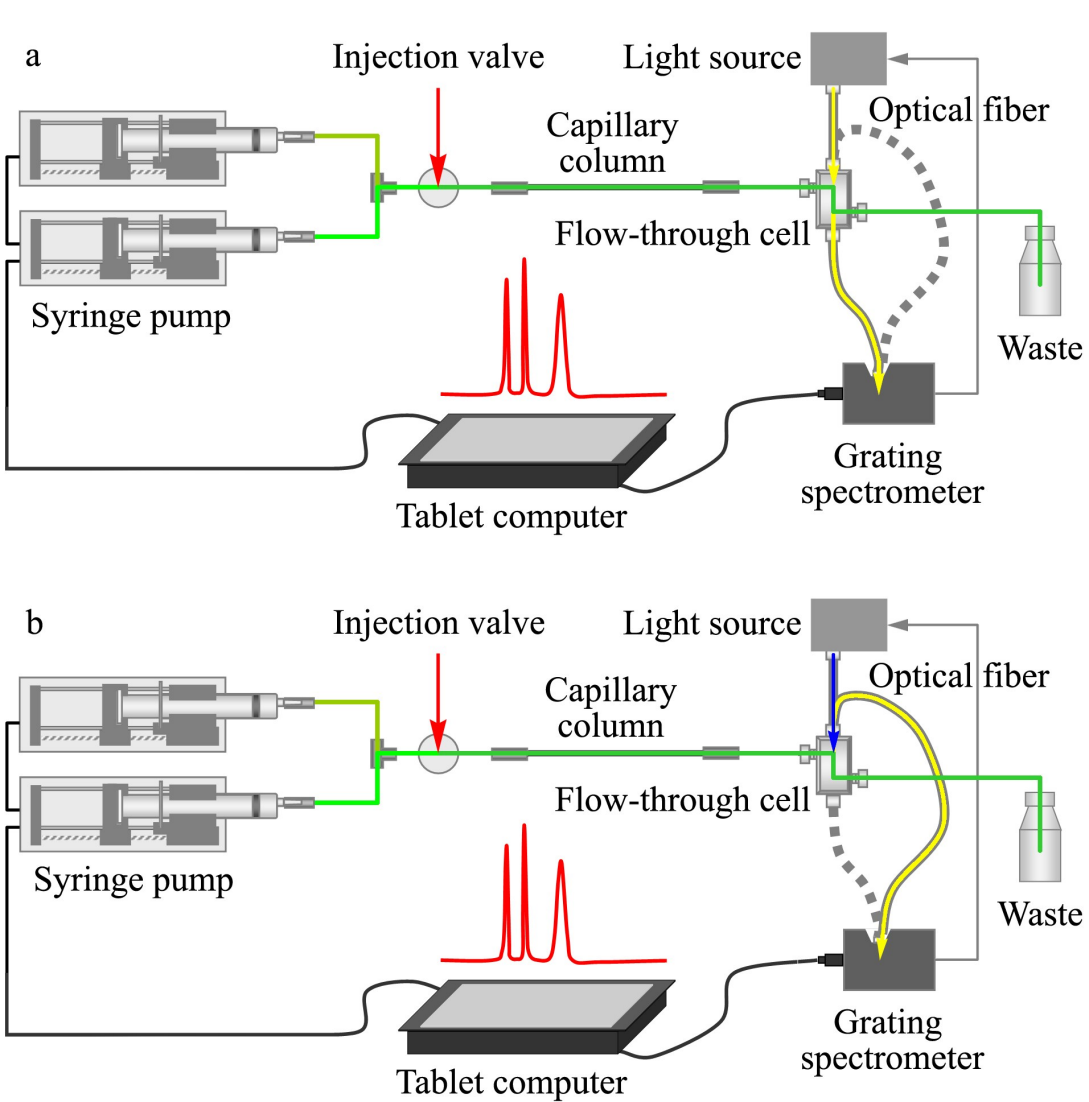
*p*-μLC结构和工作原理

## 1 *p*-μLC的整体结构设计

升级版*p*-μLC沿用了我们前期制作的*p*-μLC的基本模块化结构^[14]^,主要部件包括1)可梯度驱动流动相的注射泵组件和微量进样阀;2)毛细管聚合物整体柱和安装架;3)自主设计研制的紫外-可见吸收/荧光两用微型流通池光纤组件,以及4)小型LED/脉冲氙灯组合光源和微型光栅光谱仪^[23]^。在改进型*p*-μLC仪器研制过程中,重新设计了仪器结构及各部件的组装位置和装配方式,进一步缩小了仪器体积,增加了结构强度。改进设计的*p*-μLC采用了手提箱式整体结构(335 mm×250 mm×155 mm)。仪器分为上盖和箱体两部分,上盖内装有仪器控制-数据采集模块;箱体中装配有仪器的其他组件,包括光源模块、色谱柱-流通池模块、光谱仪模块和流路模块等。各模块的结构相对独立,分别安装在机箱底部的底架或箱体壁上,方便拆装和维护。仪器全重约8 kg。仪器各模块在机箱中的位置和结构见图2。

**图2:**
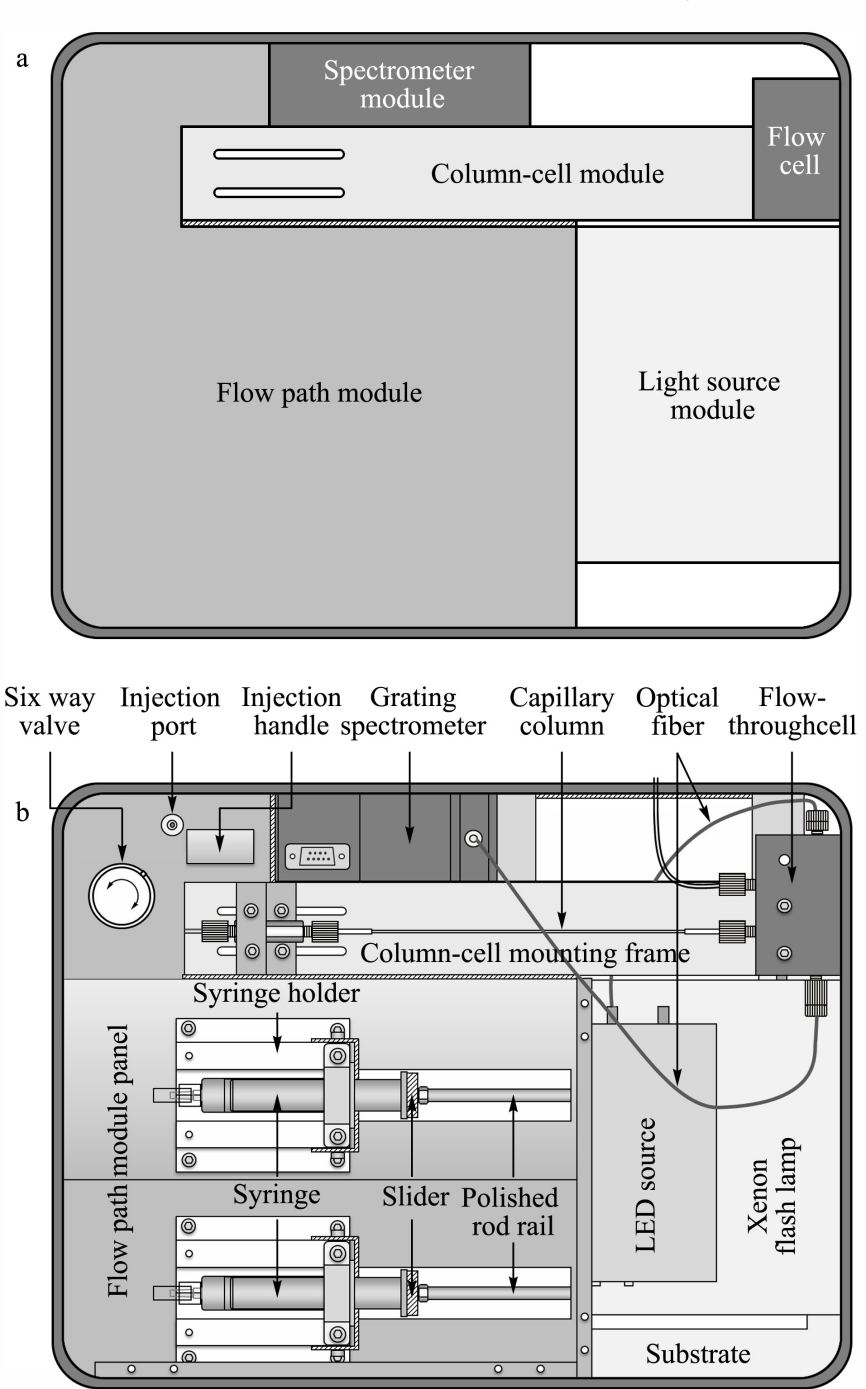
升级版*p*-μLC的(a)主要模块分布图和 (b)部件安装位置示意图(顶视图)

仪器的实物图见图3。仪器工作时,箱盖呈打开状态,一块面板遮盖住注射泵和光源部分,面板上可以放置超薄键盘,流动相补充瓶和废液瓶置于主机箱之外。

**图3:**
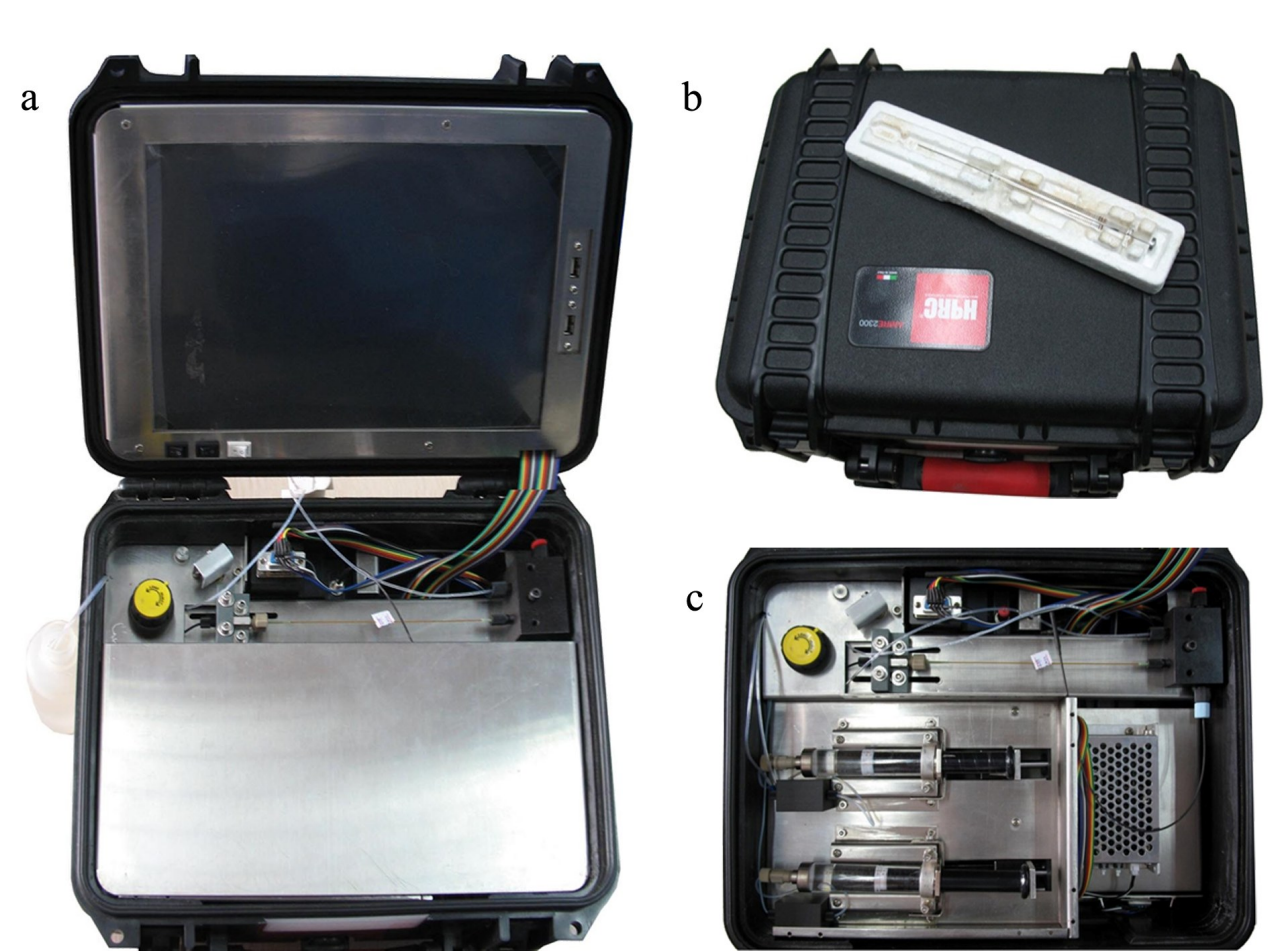
*p*-μLC的照片

## 2 *p*-μLC主要模块的设计制作

### 2.1 流路模块

针对前期研制的*p*-μLC注射泵推力不足,注射器夹持不牢固导致的流路不稳定的问题,我们重新设计制作了一种结构紧凑的大推力注射泵。为了提高注射泵的压力输出能力,采用了大扭矩步进电机和齿轮减速机以得到更大的静推力,注射泵滑块能产生的理论最大推力为760 N;设计制作的新注射器支架,可保证固定外径在15 mm以下的带锁紧接头的高压注射器(Hamilton 1010LT 10 mL, Hamilton,瑞士),实现双向推拉;优化设计注射泵结构,增加强度同时减小体积和重量;两套注射泵通过三通连接器并联,可以实现梯度洗脱。仪器注射泵可控流速范围从0.025 μL/min到5.6 mL/min,该泵在实际使用中可以达到的最大工作压强为4.5 MPa。注射泵实物图如图4所示。注射泵包括注射器支架和注射泵主体,分别安装在仪器流路模块注射器面板的上下两边。注射器支架安装在注射泵面板上,其上固定有注射器。注射泵主体安装在注射泵面板之下,其中包括步进电机、减速齿轮、丝杆、滑块等运动系统零部件。步进电机输出的扭矩经减速齿轮放大传递到具有一定螺距的丝杆上,推动滑块在两根光轴组成的轨道上平移直线运动,注射泵滑块连接注射器活塞,对注射器进行推拉动作。步进电机轴末端的截止传感器可以在滑块运行到极限位置或受到阻力过大时自动停转步进电机,以防止机械损坏或电机烧损。

**图4:**
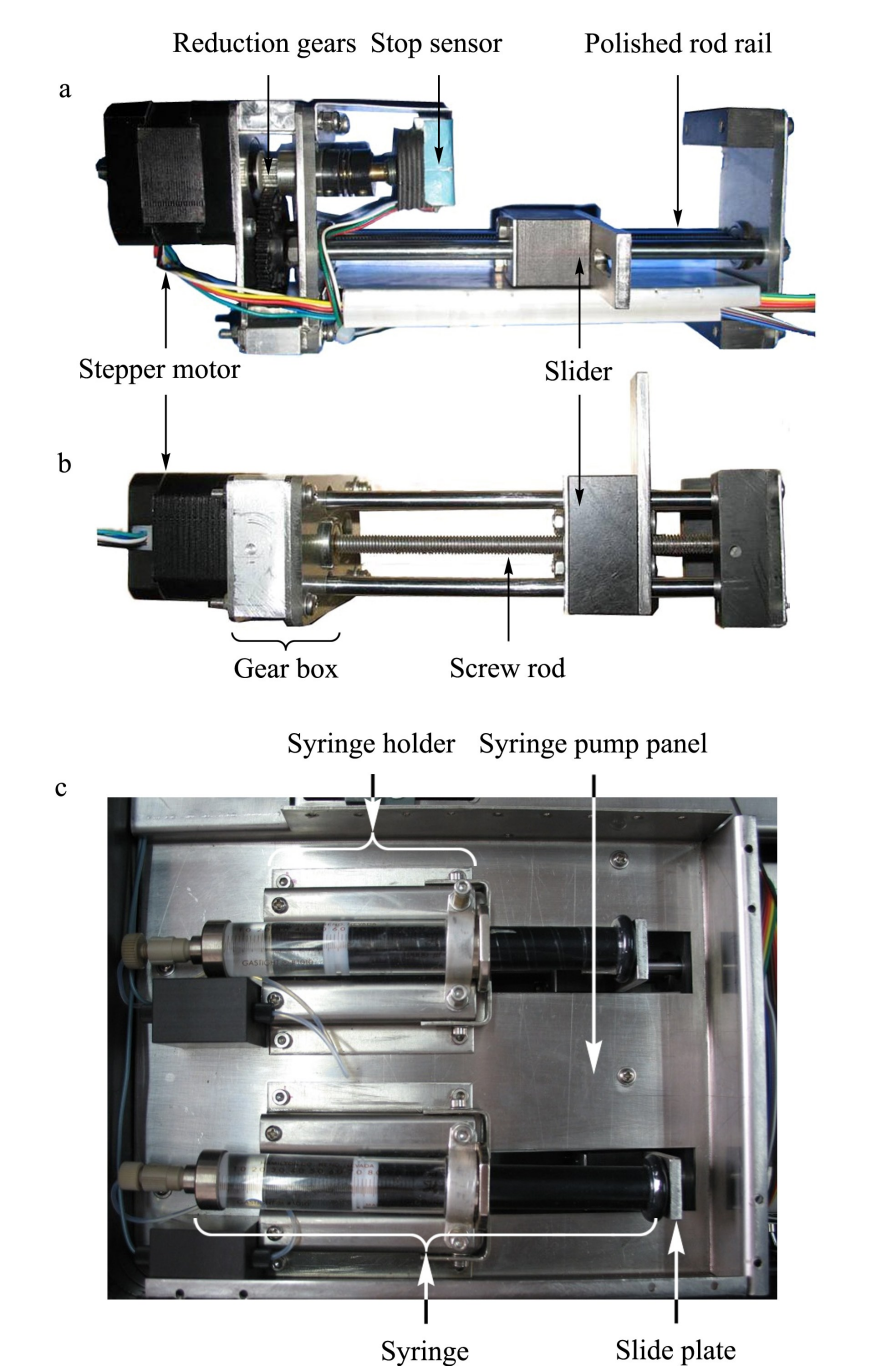
自制大推力注射泵泵体结构实物图

为了提高注射泵连续工作能力,避免频繁拆装注射器,在流路系统中通过一个位于注射泵和三通混合器之间的低压六通阀(IDEX Scientific,美国)来实现注射器再装填功能。流动相再装填组件安装在流路模块中,六通切换阀安装在流路模块面板之下,其旋钮位于进样口附近。其工作原理如图5所示。六通阀由手动操作,位于装填位置时注射器分别与对应的补充溶剂瓶联通,两套注射泵同时执行抽吸动作,可以向注射器中填充流动相;当六通阀切换到输出位置,注射器流路输出与三通混合器联通,注射泵执行推动作,输出流动相。仪器进样阀采用VICI C4-0004-.05微量进样阀(VICI,瑞士),进样体积为0.05 μL。阀体安装在流路模块面板之下,进样扳柄和独立的进样针口部件位于流路模块面板上。

**图5:**
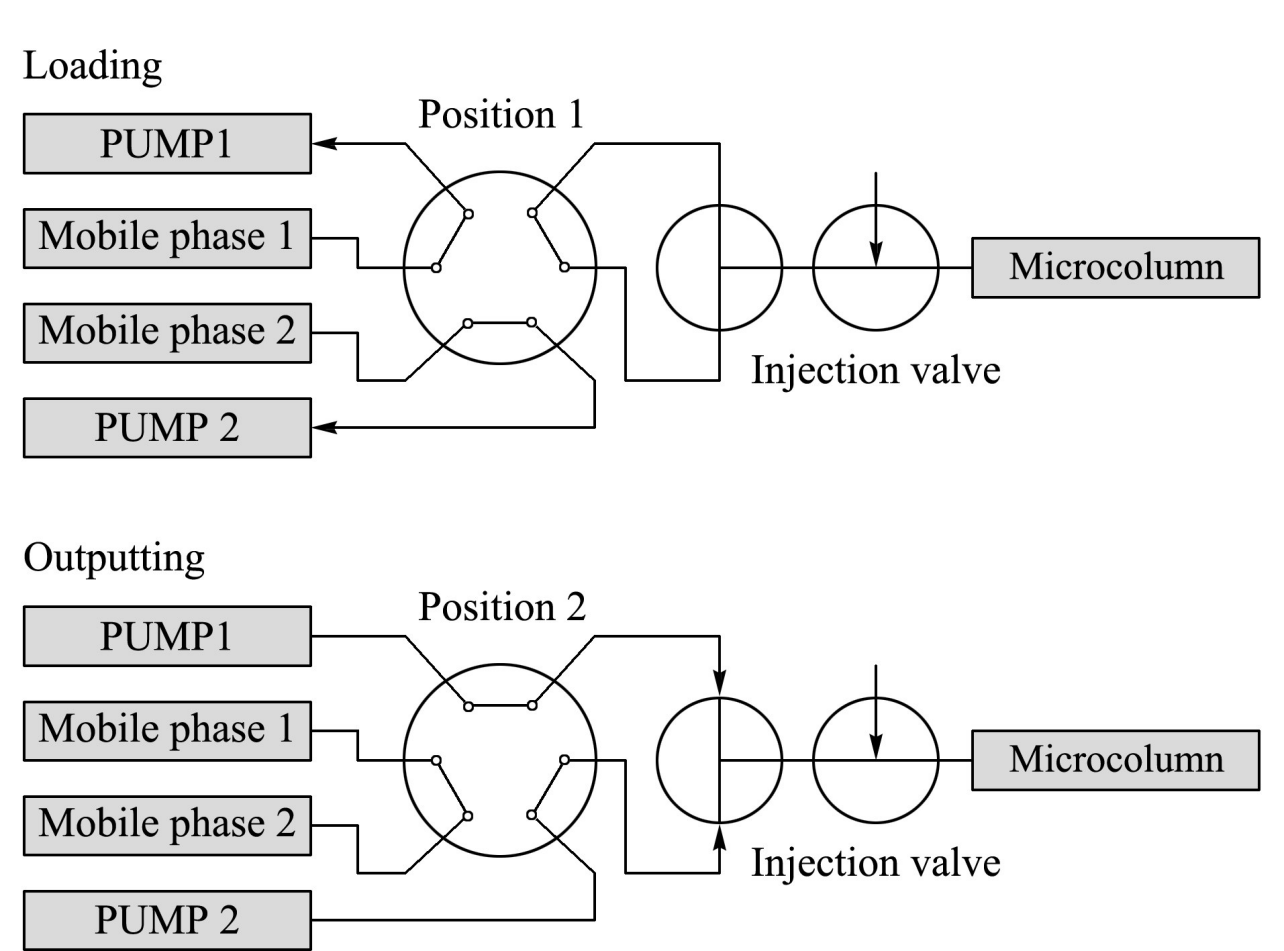
流动相再装填流路示意图

### 2.2 光源和检测器模块

为升级*p*-μLC,我们重新设计制作了光源模块。新的光源模块由小型化光纤LED光源和Ocean Optics PX-2脉冲氙灯光源组合而成。自制光纤LED光源的发光元件使用了LUXEON大功率LED(LUXEON,美国),光源中同时安装了3 W白光LED和1 W的蓝光LED,通过独立的光纤端口输出,分别作为400~680 nm可见光源和480 nm荧光激发光源,使用时可以选择白光或蓝光输出。大功率LED工作时发热量较大,因此自制光源内部设置了散热片和散热风扇。仪器使用Ocean Optics PX-2型脉冲氙灯光源作为紫外波段光源。PX-2脉冲氙灯光源是高闪光频率短弧氙灯(工作波长220~750 nm),最高工作频率220 Hz。为方便安装,PX-2脉冲氙灯除去了外壳,与自制光纤LED光源共同装配在自制支架上成为一个整体的光源模块。自制LED光纤光源和组合式光源模块实物照片见图6。

**图6:**
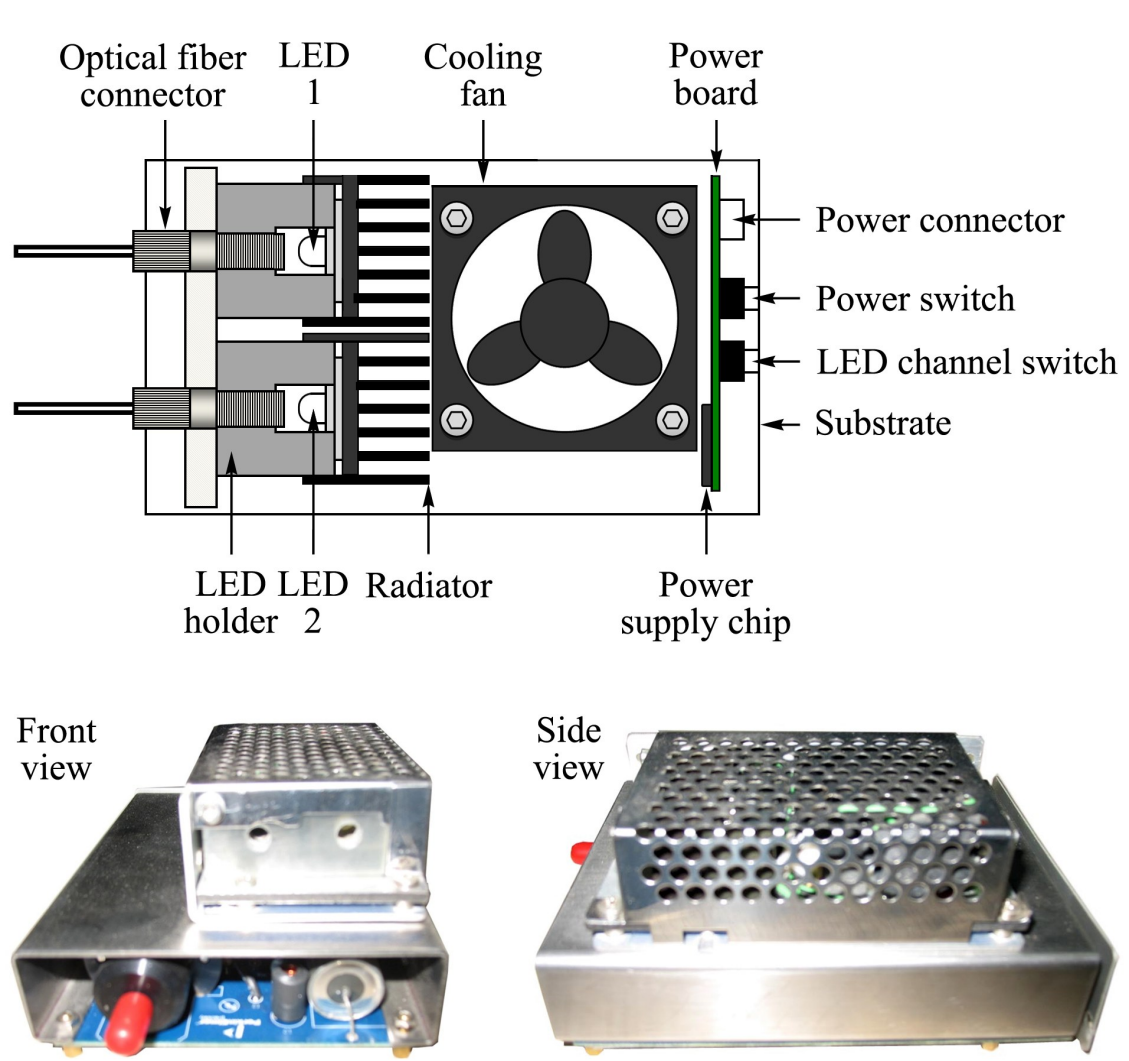
自制LED光纤光源结构和光源模块实物图

仪器检测系统的核心是光纤式紫外-可见吸收/荧光两用流通池。在流通池两端通过光纤连接光路,其中光路入口端连接一条分岔光纤,光路出口端连接的是单根光纤,分岔光纤的一支连接光源,另一支和出口单根光纤可以分别连接光栅光谱仪。使用毛细管整体柱进行分离后检测时要求流通池死体积尽量小。为减小流通池体积和尽量提高通光效率,流通池中使用了光导石英毛细管作为流通池光路部分的内腔。光导毛细管内壁石英层与外表面聚酰亚胺涂层之间存在一层折射率较低的掺杂石英层,两层石英之间的界面可以形成全反射界面;从管内射向管壁的光线入射角大于临界角23.6°时会发生全反射而折回管内,减少了流通池光路内壁对光线的吸收,从而提高了光通量,有助于提高荧光检测灵敏度。流通池中,光纤和光导毛细管之间使用自聚焦透镜作为光路耦合器件。自聚焦透镜又称为梯度变折射率透镜,其折射率分布是沿径向渐变的柱状光学透镜,此处应用其聚焦功能作为光路耦合器件,同时兼做流通池窗口。从光纤射出的发散光经过自聚焦透镜时会汇聚进入流通池,从光导毛细管射出的发散光在经过自聚焦透镜时也会汇聚进入光纤,减少了光的发散损失,提高光路耦合效率。流通池的工作原理如图7所示。在紫外-可见吸收检测模式下,来自光源的入射光由分岔光纤的一条分支进入流通池,在光导毛细管中经过样品溶液吸收后,透射光由出口的单根光纤导入光谱仪测量,得到吸光度信号;在荧光检测模式下,光源通过分岔光纤的一支与流通池一端的光路入口相连,光谱仪通过分岔光纤的另一条分支与流通池相连。从光源发出的光由分岔光纤的一支导入流通池作为激发光,激发样品溶液中的荧光性物质发出各向同性的荧光,其中与激发光呈反方向的荧光经过光路入口端的自聚焦透镜,由分岔光纤的另一支导入光谱仪,测定荧光强度,扣除杂散光背景后可得到荧光强度信号。

**图7:**
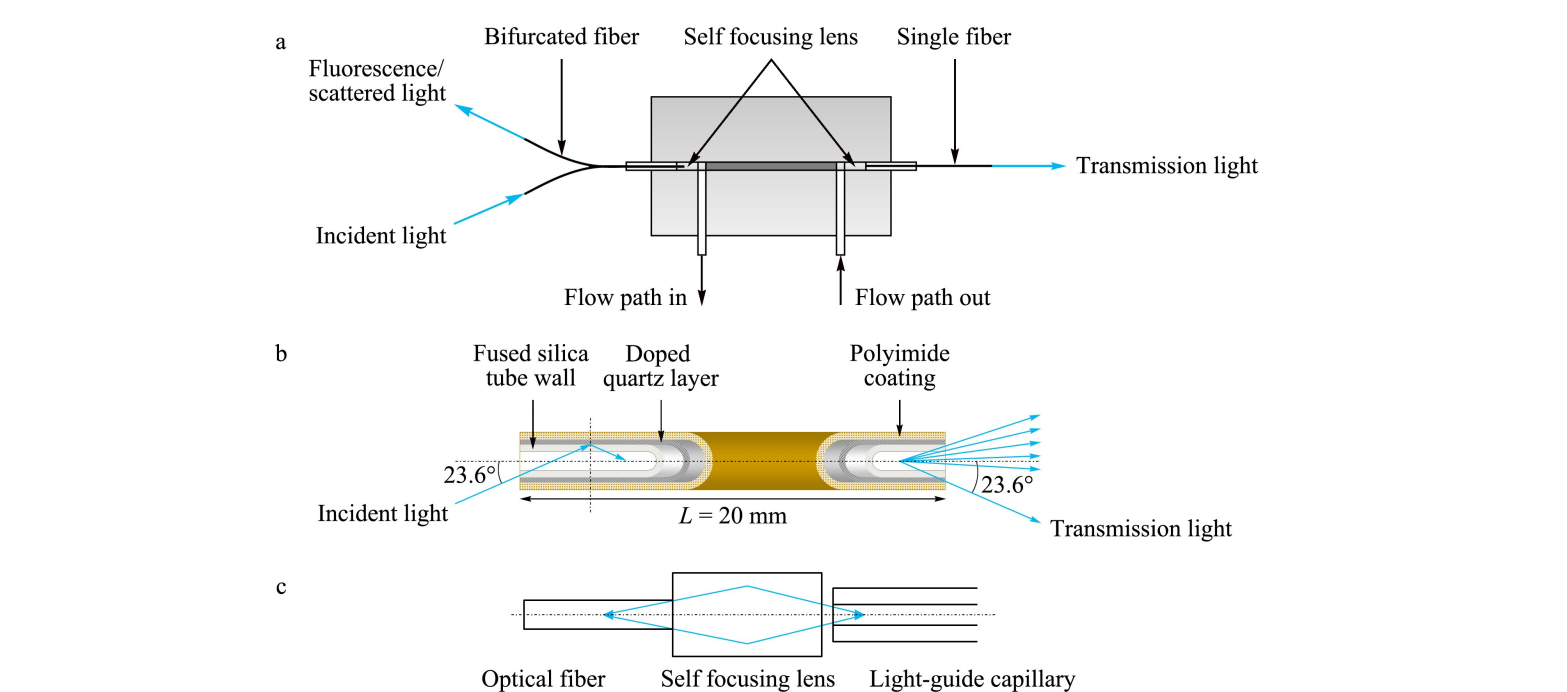
自制两用流通池的工作原理示意图

改进型*p*-μLC的研制过程中,在原有流通池基础上重新设计制作了流通池,由池体、PEEK衬管、自聚焦透镜、密封垫圈、光纤、液体流路管路等零部件构成。为解决原有流通池流路入口和出口位于池体对侧、占用空间较大且不易精确组装的问题,新的流通池中流路结构由原有的Z形改为H形,流路进口和出口位于池体的同侧,适应了更为紧凑的仪器结构要求;改进了流通池的光路/流路元件组装方式,简化了组装步骤并提高了使用稳定性;使用石英光纤制作了适配新流通池光路接口的分岔光纤和直通光纤组件。

H形流路流通池的结构如图8中所示。流通池体内沿着长轴方向有一个通孔作为光路孔,光路孔中各部件从内到外对称分布,依次为光路管、密封垫片、光路密封接头。光路密封接头由自聚焦透镜、超级无凸缘卡套和手紧接头组成。光导纤维和光导毛细管之间使用自聚焦透镜作为光路耦合器件,自聚焦透镜作为光路耦合器件的同时还起到了流通池窗口的作用,减少了光的发散损失,提高光路耦合效率。自聚焦透镜安装在内径1.8 mm的超级无凸缘卡套中,流路依靠卡套实现定位和密封。流通池的流路入口和流路出口位于流通池体的同侧,流路出入口通道与光路孔垂直,石英毛细管整体柱尾端直接插入流通池中,伸入到光路孔中以尽量减少死体积,流通池光程长为20 mm,死体积约为0.4 μL。升级版*p*-μLC采用了Ocean Optics USB2000型微型光栅光谱仪(Ocean Optics USB2000,美国)作为光学检测模块。该光谱仪以光栅为分光器件、以电荷耦合器件(CCD)为感光元件,光谱仪尺寸为89.1 mm×63.3 mm×34.4 mm,光谱检测范围200~1100 nm,光谱分辨率0.3~10 nm。光谱仪与控制计算机通过通用串行总线(USB)电缆连接,通过RS-232接口与PX-2光源进行脉冲同步。

**图8:**
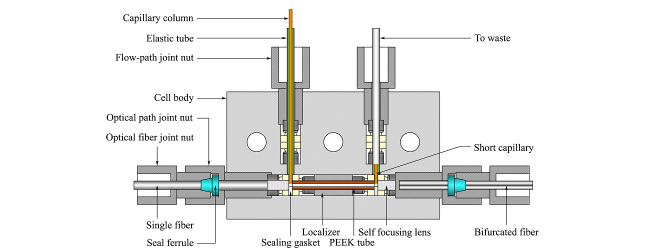
自制的H形流路流通池结构剖面图

此外,为适应升级版*p*-μLC的结构,设计制作了新的毛细管柱-流通池安装架,通过两端固定保护整体柱,可以安装长度为18~23 cm的石英毛细管整体柱,新的毛细管柱-流通池安装架是升级版*p*-μLC内部框架结构的一部分。

### 2.3 功能控制、数据采集和电源模块

仪器控制和数据采集模块包括工控主板、显示屏/触摸屏、注射泵控制单片机和USB hub等主要部件,全部安装在厚度30 mm的仪器箱体上盖中,提高了集成程度。仪器的数据采集和控制部分采用了PPC-121T/BEL平板电脑,硬件部分包括PCM-613主板、Intel Celeron M移动处理器、256M SDRAM、12寸触摸显示屏等。前期*p*-μLC上采用USB2000光谱仪配用的OOIBase32数据采集软件和自编的图形化的注射泵控制软件分别实现光谱采集和泵的控制功能。OOIBase32数据采集软件侧重于光谱数据的采集。升级版*p*-μLC仪器配备了自行编制的全新的集成化仪器操作界面软件OOLab,该软件可以实现注射泵、光源和光栅光谱仪的控制,光谱、色谱信号的实时采集、显示与记录,色谱图和色谱数据的存储、分析处理和报告输出等功能。仪器可以使用外置电源,电源变压器输出规格DC 12V 3A;仪器整体设计过程中在机箱底部预留了容纳电池组的空间,可以适时使用18650电芯锂电池组供电,便于野外现场使用。

## 3 仪器性能测试

### 3.1 检测器性能测试

使用标准溶液直接进样方式离线测试检测系统^[23]^。使用直接紫外吸收检测模式检测苯并[*α*]芘,检出限达到0.014 μg/L;在应用4-(2-吡啶偶氮)间苯二酚在线衍生检测水中的重金属元素Cd^2+^离子,检出限为10.0 μg/L;应用偶氮胂Ⅲ在线衍生检测稀土元素La^3+^离子,检出限为8.41 μg/L。应用过硫酸钾氧化-紫外吸收光度法,初步建立了测定水中总氮的实验方法,检出限为27.7 μg/L。检测系统以荧光检测模式使用时,检测荧光素的检出限为200 μg/L,检测荧光素的灵敏度较低的原因可能是由于荧光检测模式下背景散射噪声较高,仪器检测系统结构尚在进一步优化中。

### 3.2 色谱分离测试

在升级版*p*-μLC上测试其色谱分离功能。使用自制甲基丙烯酸酯C-18有机聚合物毛细管整体柱为分离柱^[20,21]^,分别在自制*p*-μLC和商品化HPLC仪器上比较了典型苯系物探针分子的分离效果(见图9)。在等度洗脱模式下,使用乙腈-水(70/30, v/v)流动相,苯系物分子都实现了基线分离,样品峰对称性良好,基线平稳;说明我们自行研制的*p*-μLC的分离检测性能可以与商品化HPLC仪器相媲美。

**图9:**
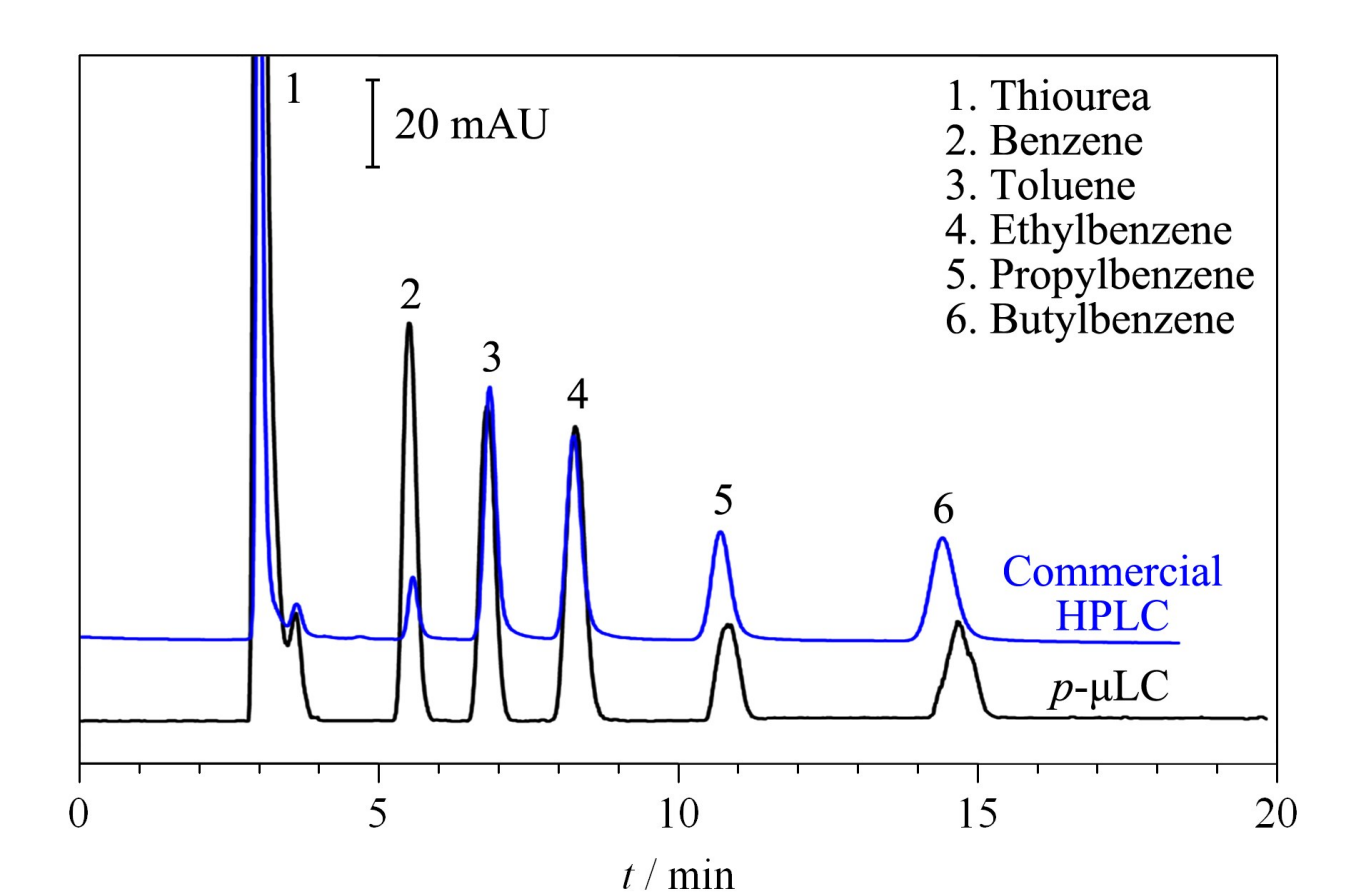
苯系物在*p*-μLC和商品化HPLC仪器上的分离谱图

## 4 结论

我们研制了一种*p*-μLC仪器。仪器包括了自主设计研制的大推力注射泵、毛细管整体柱、自制的高亮度二极管和脉冲氙灯光纤光源和改进结构的紫外-可见/荧光检测装置,可以实现恒流和梯度洗脱分离模式,并根据样品的特性可选用紫外-可见吸收或荧光检测;在具备了基本的色谱分离和检测功能的同时,实现了微型化便携式。该仪器结构紧凑,体积小,重量轻,防水、抗震,方便携带,适于野外现场监测使用。随着各种新型有机-无机杂化毛细管整体材料研究的不断进步^[21,24-26]^, *p*-μLC不仅将为野外现场监测提供便携式的检测工具,也将灵活地与各类实验室检测仪器,如质谱等,联合使用,提高工作效率。
